# Cerium Addition Improved the Dry Sliding Wear Resistance of Surface Welding AZ91 Alloy

**DOI:** 10.3390/ma11020250

**Published:** 2018-02-06

**Authors:** Qingqiang Chen, Zhihao Zhao, Qingfeng Zhu, Gaosong Wang, Kai Tao

**Affiliations:** 1 School of Materials Science & Engineering, Northeastern University, Shenyang 110819, China; neucqq@163.com; 2 Key Laboratory of Electromagnetic Processing of Materials, Ministry of Education, Northeastern University, Shenyang 110819, China; neuzhuqf@163.com (Q.Z.); quinley@126.com (G.W.); taokai092@163.com (K.T.)

**Keywords:** AZ91 magnesium alloy, surfacing weld, wear resistance, cerium

## Abstract

In this study, the effects of cerium (Ce) addition on the friction and wear properties of surface welding AZ91 magnesium alloys were evaluated by pin-on-disk dry sliding friction and wear tests at normal temperature. The results show that both the friction coefficient and wear rate of surfacing magnesium alloys decreased with the decrease in load and increase in sliding speed. The surfacing AZ91 alloy with 1.5% Ce had the lowest friction coefficient and wear rate. The alloy without Ce had the worst wear resistance, mainly because it contained a lot of irregularly shaped and coarse β-Mg_17_Al_12_ phases. During friction, the β phase readily caused stress concentration and thus formed cracks at the interface between β phase and α-Mg matrix. The addition of Ce reduced the size and amount of Mg_17_Al_12_, while generating Al_4_Ce phase with a higher thermal stability. The Al-Ce phase could hinder the grain-boundary sliding and migration and reduced the degree of plastic deformation of subsurface metal. Scanning electron microscopy observation showed that the surfacing AZ91 alloy with 1.5% Ce had a total of four types of wear mechanism: abrasion, oxidation, and severe plastic deformation were the primary mechanisms; delamination was the secondary mechanism.

## 1. Introduction

As the lightest-metal structural materials in practical applications, magnesium alloys have the advantages of a high specific stiffness and specific strength; they have been increasingly used in aerospace, military equipment, automobile manufacturing, and other fields [1,2,3,4]. During actual use, various local damage such as scratches, corrosion, wear, and tear on the surface of magnesium alloy structural parts or components are inevitable. These damages severely affect the overall performance and service life of magnesium alloy materials. A lot of resources would be consumed if all these materials with local damages were replaced. Surface welding, a traditional surface cladding technology, can be used to perform effective and quick repair of local damages such as wear on the surface of magnesium alloy equipment. Surface welding can also be used for surface strengthening of materials and rapid prototyping of parts or components [5,6]. Therefore, surface welding has great importance for extending the service life of metal equipment, thus saving money and shortening the maintenance period. In addition, owing to flexibility and simple operation as well as high repair efficiency, surface welding has very important application value in some special situations such as fast repair of weapons and equipment on the battlefield [7,8,9].

Wear is an important form of material failure and significantly affects the service status of magnesium alloy materials. Therefore, in recent years, friction and wear of magnesium alloys have gradually become a hot topic in the tribology field. Researchers have found that friction and wear of magnesium and magnesium alloys can be significantly improved by adding rare-earth elements [10,11,12,13,14]. Hidetoshi et al. [12] studied the wear of squeezed AZ91 magnesium alloy containing Y and found that a small amount of Al_2_Y phase improved the hardness and thermal stability of the alloy, thus increasing the friction and wear resistances of the alloy. Kaveh et al. [13] evaluated the effects of adding mixed rare-earth elements on the wear of as-cast AZ91 magnesium alloys and found that Al-RE phases pinned the grain boundaries during friction process and thus improved the high-temperature wear resistance of metals.

Local wear resistance performance of welding parts significantly affects the overall performance of the material. However, the friction and wear of magnesium alloys after surface welding have rarely been studied. In this study, different amounts of rare-earth element Ce were added to traditional AZ91 magnesium alloy welding wires; these welding wires were used for surface welding. The effects of adding rare-earth element Ce on the dry sliding friction and wear of surface welding magnesium alloys were evaluated, and the wear mechanism was elucidated.

## 2. Experimental Procedure

**Figure 1:**
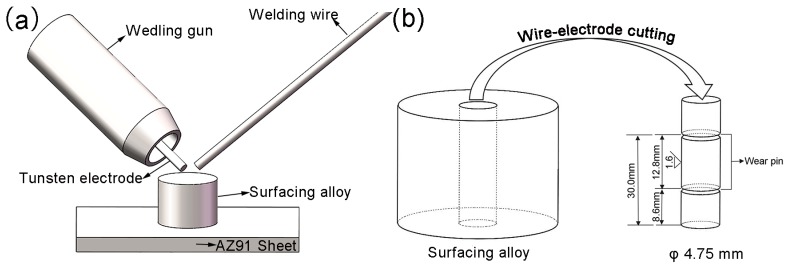
Schematic representation of (**a**) the surface welding and (**b**) the sampling location.

The diameter of magnesium alloy welding wire was 2.5 mm. A commercial as-cast magnesium alloy sheet with a thickness of 10 mm was used as the welding base. The chemical compositions of welding wire and parent metal were analyzed by spark emission spectrometer (FOUNDRY-MASTER Xpert, Oxford Instruments, Abingdon, UK). Before welding, first the oxide film on the surface of parent metal was removed by mechanical polishing, and then the surface of the parent metal was cleaned with absolute ethyl alcohol. Surface welding of parent metal surface was performed using tungsten inert gas welding; the size of welding sample was φ 20 mm × 30 mm. Figure 1a shows the schematic diagram of surface welding. The welding current was 130 A, and the voltage was 15 V. The welding was performed under 99.99% pure argon atmosphere; the flow rate of argon was 16 L/min. The surface welding samples were cut along the longitudinal section by linear cutting for mechanical polishing; then the polished surface was corroded with mixed acids (5 g picric acid, 5 mL acetic acid and 10 mL distilled water and 100 mL absolute ethyl alcohol) to make the phase more visible under the microscope. Then, the microstructure was observed by optical microscopy (OM, DMI5000M, Leica Microsystems, Wetzlar, Germany) and scanning electron microscopy (SEM, SSX-550, SHIMADZU, Kyōto, Japan) equipped with an energy dispersive spectrometer (EDS, SHIMADZU, Kyōto, Japan). The phase compositions of surface welding samples with different Ce contents were analyzed using an X-ray diffractometer (XRD, MPDDY2094, PANalytical B.V., Almelo, The Netherlands) with an accelerating voltage and current of 40 kV and 40 mA, respectively. The obtained XRD data were fitted and quantitatively analyzed using the Rietveld method [15] and GSAS analysis software (1.00, Argonne National Laboratory, Du Page, IL, USA) [16] to calculate the volume percentage of each phase. The Vickers hardness of samples was tested using a macrohardness tester (452-SVD, Wolpert, Aachen, Germany) with a load of 3 kg and loading time of 15 s, and the average of four tested hardness for each sample was taken as the final hardness.

**Figure 2:**
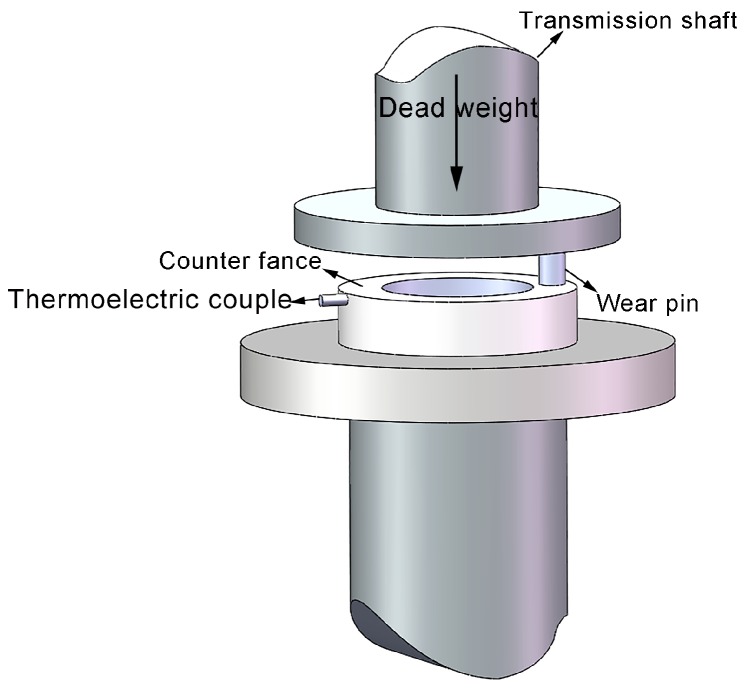
Schematic representation of surface welding the pin-on-disc experimental configuration.

Friction and wear experiments were conducted on a vertical universal friction and wear-testing machine (MMD-1, Jinan Yihua Tribology Testing Technology Co. Ltd., Jinan, China); the friction mode was pin-on-disk dry sliding friction and wear at normal temperature (~25 °C). The size of friction pin was φ 4.75 mm × 12.8 mm. To avoid the side effects of microstructural heterogeneity on experimental results, all the friction pins were taken from the central parts of surface welding samples (Figure 1b). The paired abrasive disk was made from ASTM1045 steel with ~45 HRC. In the experiments, the loads were 10 N and 20 N; the sliding velocities were 0.25 m/s, 0.5 m/s, 0.75 m/s, 1.0 m/s, and 1.25 m/s; the sliding distance was 1.5 km. Figure 2 shows the schematic of friction and wear experiment. To evaluate the effects of experimental conditions on the generated frictional heat during friction, some holes were drilled near the friction contact surface on abrasive disk, and thermocouples were embedded in the holes. Each sample was weighed before and after the experiments using an electronic analytical balance (AUX220, SHIMADZU, Kyōto, Japan), and the average of three measurements was taken as the sample weight to calculate the volume wear rate. After the experiments, the friction pins were cut along the longitudinal section, and the subsurface layer microstructure of friction surface was observed using an optical microscope. The morphologies of wear surface and wear debris were observed using an SEM.

## 3. Results and Discussion

### 3.1. Composition and Microstructure

**Figure 3:**
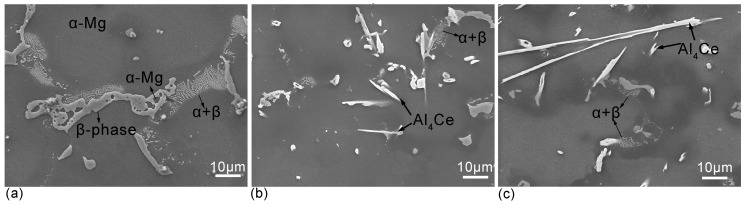
SEM micrograph of the alloys: (**a**) surfacing AZ91 alloy with 0% Ce; (**b**) surfacing AZ91 alloy with 1.5% Ce; (**c**) surfacing AZ91 alloy with 3% Ce.

The chemical compositions of welding wire and basic metal are shown in Table 1. The quite similar composition is of great benefit to investigating the effect of Ce on the microstructure and wear resistance of alloy without any disturbance from Al, Zn and Mn. Figure 3 shows the microstructure images of surface welding AZ91 alloys with different contents of Ce. Figure 3a shows that the microstructure without Ce addition is mainly composed of α-Mg matrix and reticulate and bulky irregular β-Mg_17_Al_12_ phase distributed at grain boundaries. A small amount of α-Mg was observed in the holes in the β-Mg_17_Al_12_ phase; a laminar α + β eutectic microstructure was observed at the edge of β-Mg_17_Al_12_ phase. This microstructure is similar to the solidified structure of as-cast AZ91 magnesium alloy [17,18,19]. After 1.5% Ce addition, the reticulate β-Mg_17_Al_12_ phase in the microstructure of surface welding alloy was broken into chain-like and bulky phase (Figure 3b), and its total content also decreased. Moreover, a small amount of needle-like and bulky Al_4_Ce phase with 10–20 μm size also appeared. When the content of Ce was 3%, both the size and amount of β-Mg_17_Al_12_ phase decreased (Figure 3c), and the size of Al–Ce phase increased to a length of up to 80–100 μm.

**Figure 4:**
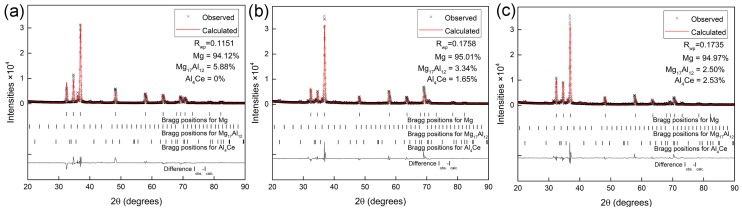
The measured and calculated X-ray diffractogram of the surfacing alloys. (**a**) surfacing AZ91 alloy with 0% Ce; (**b**) surfacing alloy with 1.5% Ce; (**c**) surfacing alloy with 3% Ce.

Figure 4 shows the XRD results of surface welding magnesium alloys; the refined curves obtained after Rietveld fitting and quantitative phase analysis are also labeled. The phase composition of surface welding AZ91 alloy without Ce is mainly composed of α-Mg and β-Mg_17_Al_12_; the volume percentage of β-Mg_17_Al_12_ is 5.88%. After the addition of 1.5% Ce, the Ce atoms combined with Al atoms to form Al_4_Ce phase, and the volume percentage of β-Mg_17_Al_12_ decreased to 3.34%. With the continuous increase in Ce content, the volume percentage of Al_4_Ce phase increased, while that of β-Mg_17_Al_12_ continuously decreased. Table 2 shows the measured macrohardness of alloys with three different compositions. The hardness of surface welding alloys gradually decreased with the increase in Ce content. This is mainly because β-Mg_17_Al_12_ has a hard brittle phase; the microhardness of this phase is significantly higher than that of Mg matrix [18]. The addition of Ce decreases the amount of β-Mg_17_Al_12_ phase, thus decreasing the macrohardness of alloy.

### 3.2. Friction and Wear Performance

**Figure 5:**
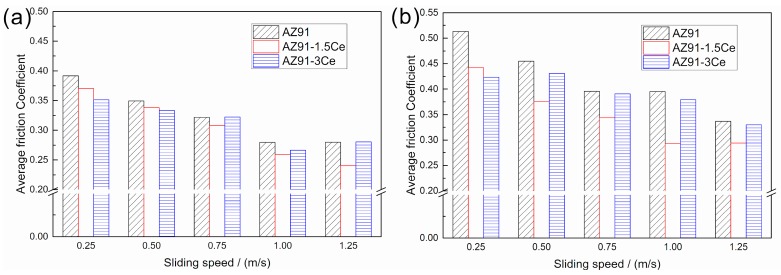
Average friction coefficient for (**a**) 10 N, (**b**) 20 N loads.

**Figure 6:**
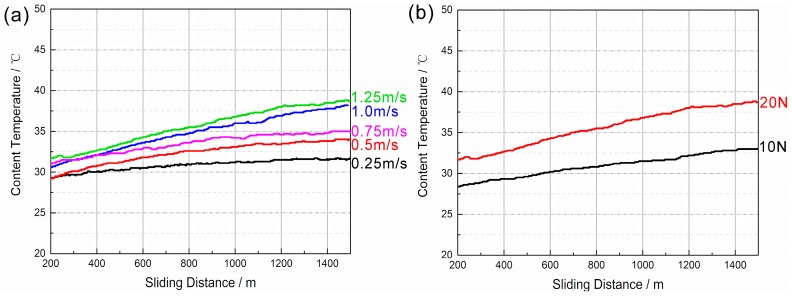
Contact temperature versus sliding distance for (**a**) normal load of 20 N and (**b**) sliding speed of 1.25 m/s.

Figure 5 shows the friction coefficients of surface welding alloys under different loads and sliding speeds. When the load is fixed, the friction coefficients of the three types of surface welding alloys showed the same trend, i.e., gradually decreased with the increase in sliding speed. This trend is similar to that of Mg-Y-Gd alloy reported by Cao et al. [20]. This is probably because a lot of friction heat is produced at the contact surface of friction pair during friction. With the increase in sliding speed, the generated heat at the contact surface gradually increases, and the degree of thermal softening of metal at the contact surface increases due to friction heat, thus facilitating the sliding and reducing the friction coefficient [21]. Because it is difficult to accurately measure the heat generated by friction, the temperature near the location of friction contact surface on abrasive disk was measured and recorded. The results are shown in Figure 6. The temperature increased with the increase in friction velocity or experimental load. This reflects the trend of frictional heat generated on friction contact surface to some extent.

In addition, when the sliding speed is fixed, although an increase in load increases the friction heat (Figure 6b), it also increases the friction coefficient of alloy (Figure 5). This can be attributed to the microscopic elastic and plastic deformation of metal surface during the friction and wear. The friction pair has mutual contact owing to microscopic bumps that share the load together, and the total contact area does not linearly correlate with the load. An increase in load makes the microscopic bumps generate a larger plastic deformation and increases the contact area, thus increasing the friction coefficient [22].

**Figure 7:**
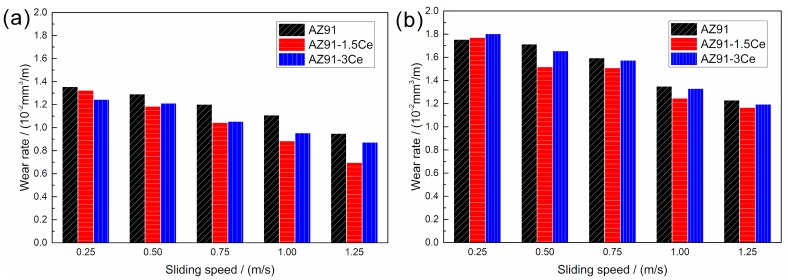
Wear rate values for (**a**) 10 N, (**b**) 20 N loads.

Figure 7 shows the wear rate of surface welding alloy under different loads and sliding speeds. When the load is fixed, an increase in sliding speed decreases the wear rate. This is probably because magnesium alloys themselves are oxidized easily. With the increase in sliding speed, the heat generated by friction increases significantly, and a thick oxide layer is formed on the magnesium alloy surface to protect the matrix [20,23,24]. When the sliding speed is fixed, an increase in load increases the wear rate. This is because an increase in load enhances the degree of plastic deformation of metal in surface layer and strengthens the microscopic cutting effects of microscopic bumps on abrasive disk on metal surface, thus increasing the wear rate.

**Figure 8:**
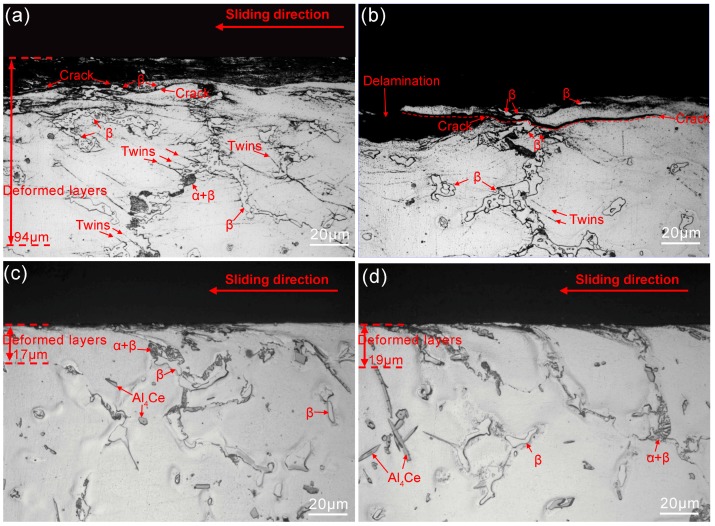
Micrographs of the subsurface of surfacing alloys after sliding under an applied load of 10 N and sliding speed of 1.25 m/s. (**a**,**b**) surfacing AZ91 alloy with 0% Ce; (**c**) surfacing AZ91 alloy with 1.5% Ce; (**d**) surfacing AZ91 alloy with 3% Ce.

**Figure 9:**
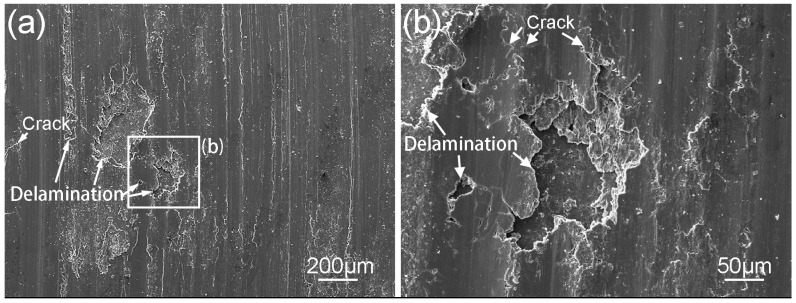
(**a**) Delamination mode of wear for surfacing AZ91 alloy tested at 1.25 m/s and 20 N and (**b**) the corresponding enlarged view of (**a**).

According to the hardness measurement results, the addition of Ce decreases the hardness of surface welding alloy. However, as shown in Figure 7, the wear rate of AZ91 surface welding alloy containing 1.5% Ce is significantly less than that of AZ91 alloy without Ce. This is not consistent with the Archard’s law [25]. Feng et al. [26,27] found that during the deformation of an as-cast AZ magnesium alloy, the interface between coarse β-Mg_17_Al_12_ and α-Mg matrix easily became the source of cracks, thus adversely affecting the mechanical properties of alloy. In addition, our previous studies [28] proved that the β-Mg_17_Al_12_ phase in surface welding AZ91 alloy can be dissolved by a solid-solution treatment, thus significantly improving the wear resistance. In our experiment, the irregularly shaped β-Mg_17_Al_12_ phase in surface welding AZ91 alloy can also cause stress concentration during friction and wear and thus induce the precipitation of abundant twin crystals surrounding β-Mg_17_Al_12_ phase, as shown in Figure 8a,b. During continuous friction, cracks may be initiated at the interface between β-Mg_17_Al_12_ and α-Mg matrix. The cracks continuously propagate to the worn surface and lead to the spalling of surface metal, thus forming spalling pits. As a result, the wear resistance of surface welding alloy is reduced by increasing the degree of delamination wear. The cracks and spalling pits in the subsurface microstructure of alloy are shown in Figure 8b. Figure 9 shows the SEM images of spalling pits on the worn surface of alloy after the friction. On the other hand, during friction and wear, large amounts of friction heat may be produced on contact surface; moreover, studies [29,30,31] have proved that β-Mg_17_Al_12_ phase has a poor thermal stability. Therefore, coarse β-Mg_17_Al_12_ phase has no significant strengthening or pinning effect on grain boundaries during the plastic deformation of surface metal. In addition, the hardness of bulky β-Mg_17_Al_12_ phase is much higher than that of matrix, and β-Mg_17_Al_12_ phase may peel off entirely and adhere to the space between the material and friction disk, thus preventing the relative sliding of friction pair and increasing the abrasive wear.

After the addition of Ce, both the size and amount of irregularly shaped coarse β-Mg_17_Al_12_ phase reduced, thus improving the friction and wear of alloy. In addition, Al_4_Ce phase formed by Ce addition is a high-temperature-resistant phase, and the thermal stability of this phase is much higher than that of β-Mg_17_Al_12_ phase. This can effectively pin the grain boundaries during sliding [13], thus hindering the sliding and migration of grain boundaries and decreasing the degree of plastic deformation of subsurface metal. As shown in Figure 8, the thickness of plastic deformation layer in surface welding alloy containing Ce is far less than that of plastic deformation layer in surface welding alloy without Ce. Within the sliding speed range 0.75–1.25 m/s, the wear rate of surface welding alloy containing 3% Ce is higher than that of surface welding AZ91 alloy containing 1.5% Ce. This is probably because a very high content of Ce leads to the formation of an over coarse Al_4_Ce phase (Figure 3).

### 3.3. Wear Mechanism

The differences in wear performance are related to different wear mechanisms of surface welding alloy under different friction conditions. In this experiment, four wear mechanisms were observed by SEM analysis. Among them, abrasive wear, oxidative wear, and severe plastic deformation are the primary wear mechanisms, and delamination wear is the secondary wear mechanism.

**Figure 10:**
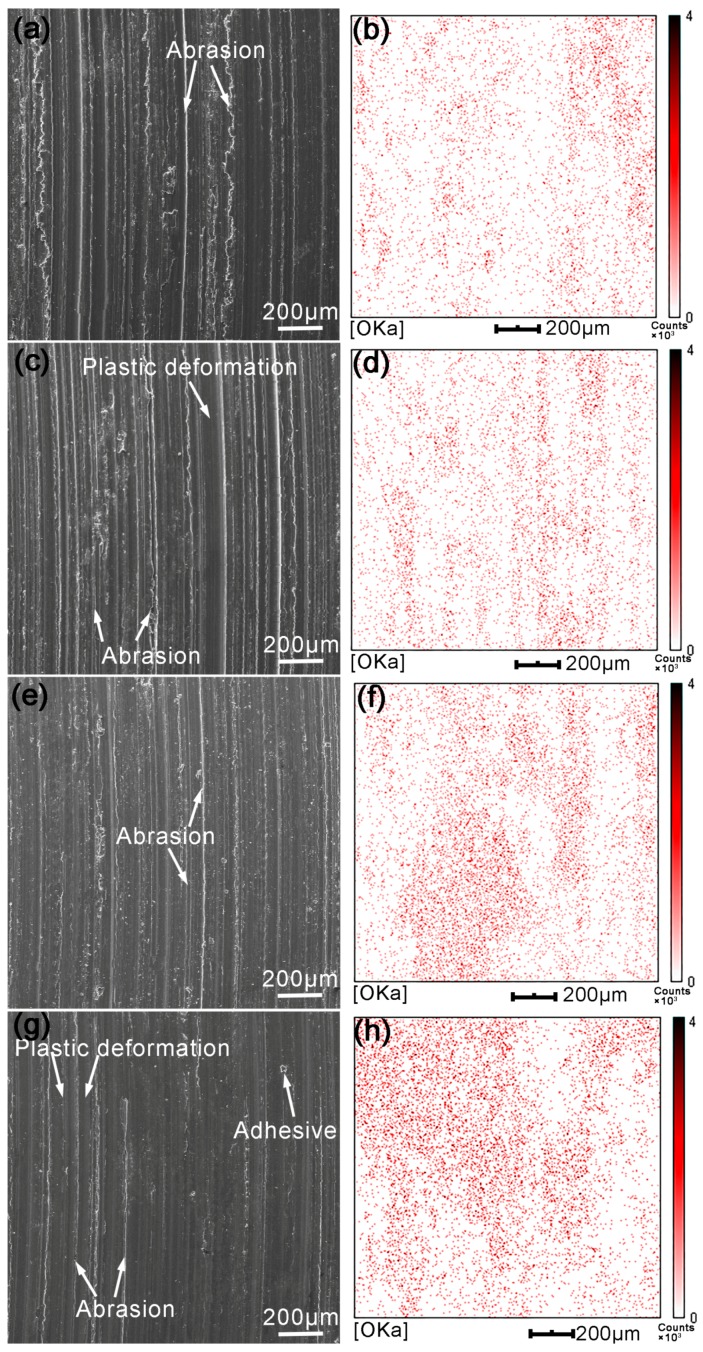
SEM micrograph and the results of EDS plane scan of the worn surface of surfacing alloys with 1.5% Ce after sliding under a sliding load of 10 N and different speeds: (**a**) 0.25 m/s; (**b**) EDS analysis of (**a**); (**c**) 0.5 m/s; (**d**) EDS analysis of (**c**); (**e**) 1.0 m/s; (**f**) EDS analysis of (**e**); (**g**) 1.25 m/s; (**h**) EDS analysis of (**g**).

Figure 10 shows the wear surface morphology and energy dispersive spectrum (EDS) area-scan analysis results of surface welding AZ91 alloy containing 1.5% Ce when the load is 10 N. Many grooves and scratches were observed on the wear surface parallel to the sliding direction. This is the main characteristic of abrasive wear, mainly caused by the cutting and plowing effects on the alloy of hard microscopic bumps on the paired abrasive disk surface and deciduous wear debris particles during friction. With the increase in sliding speed, both the depth and width of grooves and scratches gradually decreased, indicating a decrease in the degree of abrasive wear of material. Notably, when the sliding speed is low (0.25–0.5 m/s), the edges of grooves and scratches are rougher, and some edges of scratches showed a serrated shape (Figure 10a,c). With the increase in sliding speed (1.0–1.25 m/s), the grooves became shallower, and the scratch edges became flat (Figure 10e,g). The reasons for morphology change on the worn surface can be summarized as follows: When the sliding speed is low, the friction heat produced by sliding is limited, and the plasticity of magnesium alloys at room temperature is poor. Thus, the surface metal breaks easily when the microscopic bumps or abrasive particles on abrasive disk migrate along the contact surface under the action of shear stress, forming deep grooves and serrated scratches (Figure 10a,c). As the sliding speed increases, a lot of frictional heat is produced by friction, improving the plasticity of surface metal. At this moment, the metal does not break under the plowing effects of grits, but suffers plastic deformation and is squeezed to both sides of the grooves, forming shallower grooves and scratches with smoother edges (Figure 10e,g).

**Figure 11:**
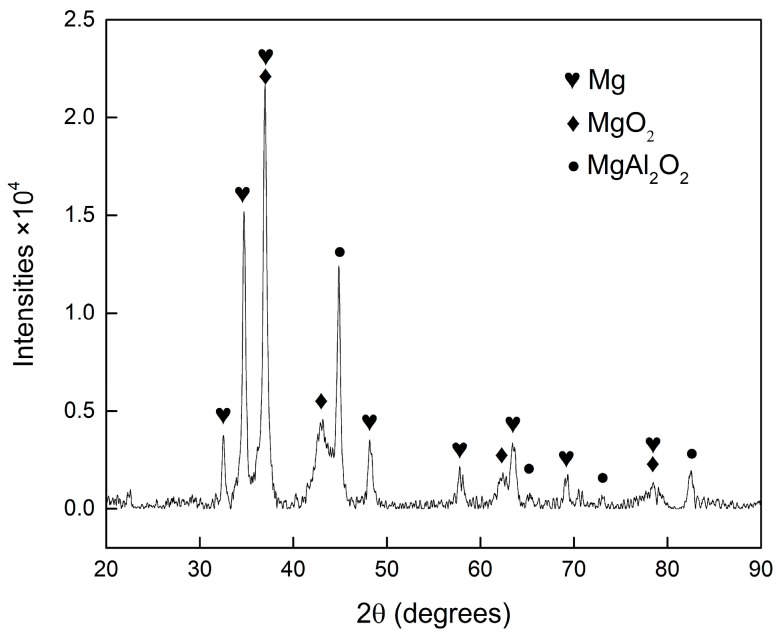
The measured X-ray diffractogram of the debris of surfacing alloys with 1.5% Ce after sliding.

The worn surface of surface welding alloy was analyzed by EDS, and the results are shown in Figure 10b,d,f,h. When the sliding speed is 0.25 m/s, a large amount of oxygen is present on the worn alloy surface (Figure 10b), indicating severe oxidative wear on the metal surface. Under this wear mechanism, the metal present on the worn surface is oxidized under the action of friction heat to form an oxidation film. With friction, the oxidation film is broken to form wear debris, while forming a new oxidation film. This cycle is repeated. With the increase in sliding speed, the produced friction heat gradually increases, and the degree of oxidative wear also gradually increases, increasing the area and thickening the oxidation layer (Figure 10d,f,h). Studies [20,23,24] showed that the oxidation film produced in the process had a protective effect on the base alloy, improving the wear resistance of the alloy. The phase composition of collected wear debris was analyzed using an XRD immediately after the friction experiment, and the results are shown in Figure 11. The XRD pattern shows a large number of oxides; this can also be used as a proof of severe oxidative wear. However, no diffraction peaks corresponding to β-Mg_17_Al_12_ and Al_4_Ce phases were observed in the abrasive wear debris. This can be attributed to their low content in the abrasive wear debris and low scanning accuracy of XRD.

**Figure 12:**
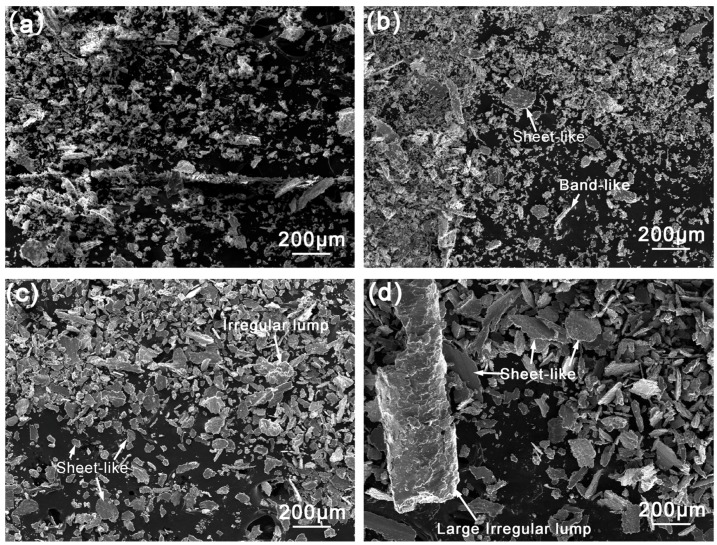
SEM micrograph of the debris of surfacing alloys with 1.5% Ce after sliding under a sliding load of 10 N and different speeds: (**a**) 0.25 m/s; (**b**) 0.5 m/s; (**c**) 1.0 m/s; (**d**) 1.25 m/s.

**Figure 13:**
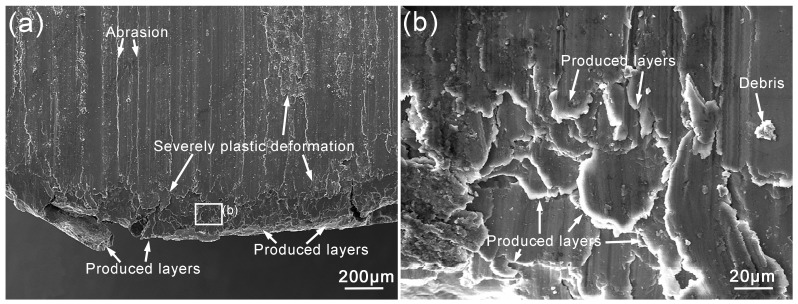
SEM images of protruded layers on the worn surface for the surfacing alloys with 1.5% Ce after sliding under 10 N and 1.25 m/s. (**a**) the protruded layers; (**b**) the corresponding enlarged view of (**a**).

Figure 12 shows the SEM images of wear debris of surface welding alloy containing 1.5% Ce. When the sliding speed is 0.25–0.5 m/s, the wear debris mainly consists of small particles with a smaller average size, as shown in Figure 12a,b. This indicates that the wear mechanism of the alloy is dominated by abrasive wear, in which the surface metal is broken to form small wear debris under the cutting effects of hard microscopic bumps and grits on abrasive disk [23,24]. When the sliding speed increases to 1.0–1.25 m/s, the size of wear debris clearly increases, and the wear debris mainly shows a flake-like morphology (Figure 12c,d). This is mainly because during friction and wear, the alloy suffers severe plastic deformation under the action of friction heat, and the surface metal is squeezed to the edges of contact surface (Figure 13), eventually spalling off to form flake-like wear debris. In addition, when the surface metal is squeezed to the edges of contact surface, it accumulates at the edges and finally spalls off to form large-size wear debris as shown in Figure 12d.

**Figure 14:**
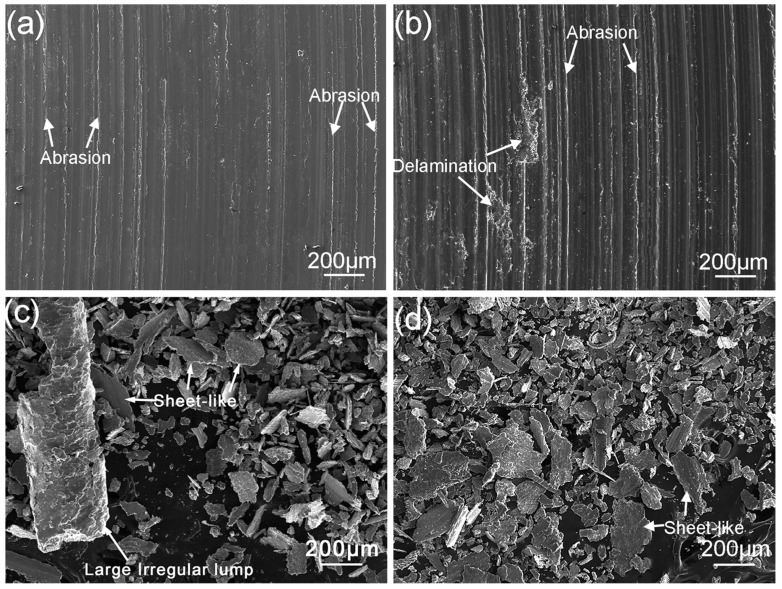
SEM micrograph of the worn surface and debris of surfacing alloys with 1.5%Ce under a sliding speed of 1.25 m/s and different loads. (**a**,**c**) 10 N; (**b**,**d**) 20 N.

Figure 14 shows the worn surface and wear debris morphology of surface welding alloy containing 1.5% Ce under different loads when the friction speed is fixed. Compared with the worn surface and wear debris morphology under a load of 10 N, the depth of grooves and scratches on the worn surface clearly increased when the load is 20 N, indicating that the abrasive wear aggravated. As a result, the wear rate of alloy under a load of 20 N increased significantly than that under a load of 10 N (Figure 7). Moreover, when the load is 20 N, some spalling pits are also observed in Figure 14b. This indicates that delamination wear occurred on the surface welding alloy containing 1.5% Ce, but the degree of wear of the alloy is less than that of the alloy without Ce (Figure 9). At the same time, because of the small size and shallow depth of pits or slight delamination wear, the wear mechanism of the material is dominated by abrasive wear and plastic deformation. By comparing the morphology of wear debris (Figure 14c,d), it was observed that the change in load had no significant effects on the morphology of wear debris.

## 4. Conclusions

In this study, the effects of adding different contents of Ce on the friction and wear of surface welding AZ91 magnesium alloys were evaluated by pin-on-disk dry sliding friction and wear tests at normal temperature. The experiment temperature was 25 °C; the experiment loads were 10 N and 20 N; the sliding speed was 0.25–1.25 m/s; the sliding distance was 1.5 km. The conclusions are as follows:

(1) Within the parameter range of our experiment, with the decrease in load and increase in sliding speed, both the friction coefficient and wear rate of three surface welding magnesium alloys with different compositions decreased. When the load was fixed, in the sliding speed range of 0.5–1.25 m/s, the surface welding AZ91 magnesium alloy with 1.5% Ce had the lowest friction coefficient and wear rate.
(2) Within the parameter range of our experiment, four wear mechanisms were observed for the surface welding AZ91 alloy containing 1.5% Ce. Among them, abrasive wear, oxidative wear, and severe plastic deformation were the primary wear mechanisms; delamination wear was the secondary wear mechanism.
(3) The friction and wear of surface welding AZ91 magnesium alloys were mainly related to their microstructures. The addition of rare-earth element Ce to the surface welding AZ91 magnesium alloy facilitated the formation of Al_4_Ce phase with a higher thermal stability, thus hindering the sliding and migration of grain boundaries and reducing the degree of plastic deformation of subsurface metal during friction. The addition of Ce element also decreased the size and amount of irregularly shaped bulky β-Mg_17_Al_12_ phase in the surface welding alloy, thus reducing its adverse effects on the friction and wear of the alloy and improving the wear resistance of magnesium alloys.

## Figures and Tables

**Table 1:** Chemical compositions of welding wires and basic metal (wt %).

Sample	Al	Zn	Mn	Ce	Fe	Si	Ni	Cu	Mg
AZ91	8.64	0.90	0.28	—	0.0051	0.0140	0.0057	≤0.0020	Bal.
AZ91 + 1.5Ce	8.83	0.89	0.22	1.40	0.0055	0.0125	0.0065	≤0.0020	Bal.
AZ91 + 3Ce	8.66	0.82	0.30	3.05	0.0050	0.0135	0.0085	≤0.0020	Bal.
Basic metal	8.95	0.71	0.33	—	0.0169	0.0107	0.0051	≤0.0020	Bal.

**Table 2:** Microhardness of the tested surfacing alloys.

Alloy	Macrohardness HV
Surfacing AZ91 alloy	69.0 ± 2.1
Surfacing AZ91 alloy with 1.5%Ce	63.7 ± 1.7
Surfacing AZ91 alloy with 3%Ce	59.1 ± 1.9
